# Supplementary material for: Flexible motor sequence generation during stereotyped escape responses
Source: eLife. 2020 Jun 5;9:e56942. doi: 10.7554/eLife.56942 (PMC7338056; doi:10.7554/eLife.56942)
Supplement: Supplementary file 1. [file elife-56942-supp1.docx]

**Supplementary File 1. Strains information**

| **Figure** | **Panels** | ***C. elegans*: Strains** | **Identifier** | **Source** |
| --- | --- | --- | --- | --- |
|  |  | *N2* |  | Shouhong Guang Lab |
|  |  | *lite-1(ce314)X* |  | CGC |
| Figure 1 | Figure 1C. | *lite-1(ce314)X; zfIs18[Pmec-4::ChR2::YFP; lin-15+]* | QW373 | Alkema Lab |
|  | Figure 1D. | *N2* |  | Shouhong Guang Lab |
| Figure 2 | Figure 2B-C. | *wenIs0010[Pinx-1::GCaMP6; Punc-122::GFP]; wenEx0769[Pinx-1::mCherry; Punc-122::RFP]* | WEN0769 | This paper |
|  | Figure 2D. | *wenEx0104[Pnpr-9::Chrimson::mCherry; Plin-44::GFP]* | WEN0104 | This paper |
|  | Figure 2D. | *pha-1(e2123)III; lite-1(ce314)X; zfIs18[Pmec-4::ChR2::YFP; lin-15+]; wenIs0125[Pnpr-9::Arch::RFP + pBX(pha-1(+)]* | WEN0127 | This paper |
|  | Figure 2D-F. | *lite-1(ce314)X; zfIs18[Pmec-4::ChR2::YFP; lin-15+]* | QW373 | Alkema Lab |
|  | Figure 2E-F. | *lite-1(ce314)X; zfIs18[Pmec-4::ChR2::YFP; lin-15+]; wenEx0866[Pnpr-9::TWK-18(gf)::mCherry; Plin-44::GFP]* | WEN0866 | This paper |
| Figure 3 | Figure 3A,C,E,F. | *wenEx0567[Pnpr-9::Chrimson::mCherry; Plin-44::GFP; Plim-4(-3328--2174)v1p1::GCaMP6::wCherry]* | WEN0567 | This paper |
|  | Figure 3C. | *inx-1(tm3524) unc-9(fc16) unc-7(e5)X* | ZM9947 | Zhen Lab |
|  | Figure 3C. | *inx-1(tm3524) unc-9(fc16) unc-7(e5)X; wenEx0612[Pnpr-9::Chrimson::mCherry; Plin-44::GFP; Plim-4(-3328--2174)v1p1::GCaMP6::wCherry]* | WEN0612 | This paper |
|  | Figure 3C. | *wenEx0567[Pnpr-9::Chrimson::mCherry; Plin-44::GFP; Plim-4(-3328--2174)v1p1::GCaMP6::wCherry]; wenEx0816[Plim-4(-3328--2174)v1p1::Arch::wCherry; Punc-122::RFP]* | WEN0816 | This paper |
|  | Figure 3D. | *inx-1(tm3524)X* | N/A | Shohei Mitani Lab |
|  | Figure 3D. | *wenEx0119[Pnpr-9::Chrimson::mCherry; Plin-44::GFP]* | WEN0851 | This paper |
|  | Figure 3D. | *inx-1(tm3524)X; wenEx0119[Pnpr-9::Chrimson::mCherry; Plin-44::GFP]* | WEN0119 | This paper |
|  | Figure 3D. | *inx-1(tm3524)X; wenEx0119[Pnpr-9::Chrimson::mCherry; Plin-44::GFP]; wenEx0826[Pnpr-9::inx-1a::UrSL::GFP; Pnpr-9::inx-1b::UrSL::GFP; Punc-122::RFP]* | WEN0826 | This paper |
| Figure 4 | Figure 4A. | *wenIs0602[Pnpr-9::Chrimson::mCherry; Plin-44::GFP]* | WEN0602 | This paper |
|  | Figure 4A. | *eat-4(ky5)III* | MT6308 | CGC |
|  | Figure 4A. | *eat-4(ky5)III; wenIs0602[Pnpr-9::Chrimson::mCherry; Plin-44::GFP]* | WEN0619 | This paper |
|  | Figure 4A. | *eat-4(ky5)III; wenIs0602[Pnpr-9::Chrimson::mCherry; Plin-44::GFP]; wenEx0842[Pnpr-9::eat-4::mCherry; Punc-122::GFP]* | WEN0842 | This paper |
|  | Figure 4A. | *wenIs0602[Pnpr-9::Chrimson::mCherry; Plin-44::GFP]; wenEx0867[Pnpr-9::TeTx::UrSL::GFP; Punc-122::RFP]* | WEN0867 | This paper |
|  | Figure 4B. | *wenEx0121[Pnpr-9::Chrimson::mCherry; Plin-44::GFP]* | WEN0852 | This paper |
|  | Figure 4B. | *avr-14(ad1305)I; avr-15(vu227) glc-1(pk54)V* | DA1316 | CGC |
|  | Figure 4B. | *avr-14(ad1305)I; avr-15(vu227) glc-1(pk54)V; wenEx0121[Pnpr-9::Chrimson::mCherry; Plin-44::GFP]* | WEN0121 | This paper |
|  | Figure 4B. | *avr-14(ad1305)I; avr-15(vu227) glc-1(pk54)V; wenEx0121[Pnpr-9::Chrimson::mCherry; Plin-44::GFP]; wenEx0827[Psto-3::avr-15a::UrSL::GFP; Psto-3::avr-15b::UrSL::GFP; Psto-3::avr-15c::UrSL::GFP; Punc-122::RFP]* | WEN0827 | This paper |
|  | Figure 4B. | *avr-14(ad1305)I; avr-15(vu227) glc-1(pk54)V; wenEx0121[Pnpr-9::Chrimson::mCherry; Plin-44::GFP]; wenEx0791[Pttx-3::glc-1::UrSL::GFP; Punc-122::RFP]* | WEN0791 | This paper |
|  | Figure 4B. | *avr-14(ad1305)I; avr-15(vu227) glc-1(pk54)V; wenEx0121[Pnpr-9::Chrimson::mCherry; Plin-44::GFP]; wenEx0825[Pttx-3::avr-14::UrSL::GFP; Punc-122::RFP]* | WEN0825 | This paper |
|  | Figure 4C. | *wenEx0680[Pavr-15::GFP; Psto-3::mCherry; Plin-44::GFP]* | WEN0680 | This paper |
|  | Figure 4C. | *wenEx0716[Pavr-14::GFP; Pttx-3::wCherry; Plin-44::GFP]* | WEN0716 | This paper |
|  | Figure 4C. | *wenEx0777[Pglc-1::GFP; Psra-11::wCherry; Plin-44::GFP]* | WEN0777 | This paper |
|  | Figure 4D. | *lite-1(ce314)X; wenEx0869[Pnpr-9::Chrimson::mCherry; Psto-3::GCaMP6::2*NLS::mCardinal; Plin-44::GFP]* | WEN0869 | This paper |
|  | Figure 4D. | *eat-4(ky5)III; wenEx0874[Pnpr-9::Chrimson::mCherry; Psto-3::GCaMP6::2*NLS::mCardinal; Plin-44::GFP]* | WEN0874 | This paper |
|  | Figure 4E. | *lite-1(ce314)X; wenEx0803[Psto-3::iGluSnFR; Pnpr-9::Chrimson::mCherry; Plin-44::GFP]* | WEN0803 | This paper |
|  | Figure 4E. | *eat-4(ky5)III; wenEx0872[Psto-3::iGluSnFR; Pnpr-9::Chrimson::mCherry; Plin-44::GFP]* | WEN0872 | This paper |
| Figure 5 | Figure 5A. | *wenIs0172[Psto-3::GCaMP6::2*NLS::mCardinal; Plin-44::GFP]* | WEN0172 | This paper |
|  | Figure 5B. | *wenEx0173[Psto-3::Chrimson::mCherry; Plin-44::GFP]* | WEN0173 | This paper |
|  | Figure 5B. | *wenEx0006[Psto-3::Arch::wCherry; Pmyo-2::GFP]* | WEN0006 | This paper |
|  | Figure 5C. | *lite-1(ce314)X; zfIs18[Pmec-4::ChR2::YFP; lin-15+]; wenEx0031[Psto-3::Arch::wCherry; Plin-44::GFP]* | WEN0031 | This paper |
|  | Figure 5D-E. | *wenEx0567[Pnpr-9::Chrimson::mCherry; Plin-44::GFP; Plim-4(-3328--2174)v1p1::GCaMP6::wCherry]* | WEN0567 | This paper |
|  | Figure 5D-E. | *wenEx0567[Pnpr-9::Chrimson::mCherry; Plin-44::GFP; Plim-4(-3328--2174)v1p1::GCaMP6::wCherry]; wenEx0840[Psto-3::miniSOG::UrSL::wCherry; Punc-122::RFP]* | WEN0840 | This paper |
|  | Figure 5F. | *lite-1(ce314)X; zfIs18[Pmec-4::ChR2::YFP; lin-15+]* | QW373 | Alkema Lab |
|  | Figure 5F. | *lite-1(ce314)X; zfIs18[Pmec-4::ChR2::YFP; lin-15+]; wenEx0471[Psto-3::miniSOG::UrSL::wCherry(30ng/ul); lin-44::GFP]* | WEN0471 | This paper |
|  | Figure 5F. | *lite-1(ce314)X; zfIs18[Pmec-4::ChR2::YFP; lin-15+]; wenEx0477[Psto-3::TeTx::UrSL::GFP; lin-44::GFP]* | WEN0477 | This paper |
| Figure 6 | Figure 6A-B. | *lite-1(ce314)X; zfIs88[Plim-4(-3328--2174)::Arch::GFP; lin-15+]* | QW889 | Alkema Lab |
|  | Figure 6B. | *lite-1(ce314)X; zfIs112[Pnpr-9::ChR2::GFP]* | QW1097 | Alkema Lab |
|  | Figure 6B. | *lite-1(ce314)X; zfIs112[Pnpr-9::ChR2::GFP]; wenEx0843[Plim-4(-3328--2174)v1p1::Arch::wCherry; Plin-44::GFP]* | WEN0843 | This paper |
|  | Figure 6C. | *lite-1(ce314)X; zfIs18[Pmec-4::ChR2::YFP; lin-15+]; wenEx0509[Plim-4(-3328--2174)v1p1::miniSOG::UrSL::wCherry; Plin-44::GFP]* | WEN0509 | This paper |
|  | Figure 6C. | *N2* |  | Shouhong Guang Lab |
| Figure 7 | Figure 7C,E. | *lite-1(ce314)X; zfIs18[Pmec-4::ChR2::YFP; lin-15+]* | QW373 | Alkema Lab |
| Figure 1—figure supplement 1 | Supplement 1A,B. | *lite-1(ce314)X; zfIs18[Pmec-4::ChR2::YFP; lin-15+]* | QW373 | Alkema Lab |
| Figure 2—figure supplement 1 | Supplement 1B. | *zfIs146[Pnmr-1::NLS::wCherry::UrSL::GCaMP6; Plim-4(-3328--2174)::NLS::wCherry::UrSL::GCaMP6; Plgc-55(-120-773)::NLS::wCherry::UrSL::GCaMP6; Pnpr-9::NLS::wCherry::UrSL::GCaMP6]* | QW1520 | Alkema Lab |
|  | Supplement 1C. | *wenIs0010[Pinx-1::GCaMP6; Punc-122::GFP]; wenEx0769[Pinx-1::mCherry; Punc-122::RFP]* | WEN0769 | This paper |
|  | Supplement 1D. | *lite-1(ce314)X; zfIs112[Pnpr-9::ChR2::GFP]* | QW1097 | Alkema Lab |
|  | Supplement 1E-G. | *lite-1(ce314)X; zfIs9[Ptdc-1::ChR2::GFP; lin-15+]* | QW910 | Alkema Lab |
|  | Supplement 1F. | *pha-1(e2123)III; lite-1(ce314)X; zfIs9[Ptdc-1::ChR2::GFP; lin-15+]; wenIs0125[Pnpr-9::Arch::RFP + pBX(pha-1(+)];* | WEN0021 | This paper |
|  | Supplement 1F. | *wenEx0104[Pnpr-9::Chrimson::mCherry; Plin-44::GFP]* | WEN0104 | This paper |
|  | Supplement 1F. | *wenEx0137[Ptdc-1::Arch::GFP; Pnpr-9::Chrimson::mCherry; Plin-44::GFP]* | WEN0137 | This paper |
|  | Supplement 1G. | *lite-1(ce314)X; zfIs9[Ptdc-1::ChR2::GFP; lin-15+]; yxEx1256[Pinx-1::TWK-18(gf)::mCherry; Punc-122::RFP]* | WEN0023 | This paper |
|  | Supplement 1H-I. | *lite-1(ce314)X; zfIs18[Pmec-4::ChR2::YFP; lin-15+]; wenEx0638[Pnpr-9::PH-miniSOG::UrSL::wCherry; Punc-122::RFP]* | WEN0638 | This paper |
| Figure 3—figure supplement 1 | Supplement 1A,B,C,F. | *wenEx0567[Pnpr-9::Chrimson::mCherry; Plin-44::GFP; Plim-4(-3328--2174)v1p1::GCaMP6::wCherry]* | WEN0567 | This paper |
|  | Supplement 1D. | *inx-1(tm3524) unc-9(fc16) unc-7(e5)X; wenEx0612Pnpr-9::Chrimson::mCherry; Plin-44::GFP; Plim-4(-3328--2174)v1p1::GCaMP6::wCherry]* | WEN0612 | This paper |
|  | Supplement 1E. | *wenEx0567[Pnpr-9::Chrimson::mCherry; Plin-44::GFP; Plim-4(-3328--2174)v1p1::GCaMP6::wCherry]; wenEx0816[Plim-4(-3328--2174)v1p1::Arch::wCherry; Punc-122::RFP]* | WEN0816 | This paper |
| Figure 4—figure supplement 1 | Supplement 1B. | *wenEx0104[Pnpr-9::Chrimson::mCherry; Plin-44::GFP]* | WEN0104 | This paper |
|  | Supplement 1B. | *eat-4(ad572); wenEx0132[Pnpr-9::Chrimson::mCherry; Plin-44::GFP]* | WEN0132 | This paper |
|  | Supplement 1B. | *eat-4(ad572); wenEx0132[Pnpr-9::Chrimson::mCherry; Plin-44::GFP]; wenEx0572[Pnpr-9::eat-4::mCherry; Pmyo-2::GFP]* | WEN0572 | This paper |
|  | Supplement 1B. | *eat-4(ky5)III; wenEx0599[Pnpr-9::Chrimson::mCherry; Plin-44::GFP]* | WEN0599 | This paper |
|  | Supplement 1B. | *eat-4(ky5)III; wenEx0599[Pnpr-9::Chrimson::mCherry; Plin-44::GFP]; wenEx0643[Pnpr-9::eat-4::mCherry; Pmyo-2::GFP]* | WEN0643 | This paper |
|  | Supplement 1B. | *eat-4(ad819)III; wenEx0607[Pnpr-9::Chrimson::mCherry; Plin-44::GFP]* | WEN0607 | This paper |
|  | Supplement 1B. | *eat-4(ad819)III; wenEx0607[Pnpr-9::Chrimson::mCherry; Plin-44::GFP]; wenEx0645[Pnpr-9::eat-4::mCherry; Pmyo-2::GFP]* | WEN0645 | This paper |
|  | Supplement 1B. | *avr-14(ad1302)I; wenEx0122[Pnpr-9::Chrimson::mCherry; Plin-44::GFP]* | WEN0122 | This paper |
|  | Supplement 1B. | *avr-15(ad1051)V; wenEx0123[Pnpr-9::Chrimson::mCherry; Plin-44::GFP]* | WEN0123 | This paper |
|  | Supplement 1B. | *glc-1(pk54)V; wenEx0565[Pnpr-9::Chrimson::mCherry; Plin-44::GFP]* | WEN0565 | This paper |
|  | Supplement 1B. | *avr-14(ad1302)I; avr-15(ad1051)V; wenEx0568[Pnpr-9::Chrimson::mCherry; Plin-44::GFP]_strain 1* | WEN0568 | This paper |
|  | Supplement 1B. | *avr-14(ad1302)I; avr-15(ad1051)V; wenEx0639[Pnpr-9::Chrimson::mCherry; Plin-44::GFP]_strain 2* | WEN0639 | This paper |
|  | Supplement 1B. | *avr-15(vu227) glc-1(pk54)V; wenEx0569[Pnpr-9::Chrimson::mCherry; Plin-44::GFP]* | WEN0569 | This paper |
|  | Supplement 1B. | *avr-14(ad1302)I; glc-1(pk54)V; wenEx0570[Pnpr-9::Chrimson::mCherry; Plin-44::GFP]* | WEN0570 | This paper |
|  | Supplement 1B. | *eat-4(ky5)III* | MT6308 | CGC |
|  | Supplement 1B. | *eat-4(ad572)III* | DA572 | CGC |
|  | Supplement 1B. | *eat-4(ad819)III* | DA819 | Zhen Lab |
|  | Supplement 1B. | *avr-14(ad1302)I* | ST1724 | Lijun Kang Lab |
|  | Supplement 1B. | *avr-15(ad1051)V* | ST152 | Lijun Kang Lab |
|  | Supplement 1B. | *glc-1(pk54)V* | NL704 | Zhen Lab |
|  | Supplement 1B. | *avr-14(ad1302)I; avr-15(ad1051)V* | DA1302 | CGC |
|  | Supplement 1B. | *avr-15(vu227) glc-1(pk54)V* | DA1370 | CGC |
|  | Supplement 1B. | *avr-14(ad1302)I; glc-1(pk54)V* | DA1384 | CGC |
|  | Supplement 1C. | *lite-1(ce314)X; wenEx0869[Pnpr-9::Chrimson::mCherry; Psto-3::GCaMP6::2*NLS::mCardinal; Plin-44::GFP]* | WEN0869 | This paper |
|  | Supplement 1C. | *eat-4(ky5)III; wenEx0874[Pnpr-9::Chrimson::mCherry; Psto-3::GCaMP6::2*NLS::mCardinal; Plin-44::GFP]* | WEN0874 | This paper |
| Figure 4—figure supplement 2 | Supplement 2A. | *lite-1(ce314)X; wenEx0803[Psto-3::iGluSnFR; Pnpr-9::Chrimson::mCherry; Plin-44::GFP]* | WEN0803 | This paper |
|  | Supplement 2A. | *eat-4(ky5)III; wenEx0872[Psto-3::iGluSnFR; Pnpr-9::Chrimson::mCherry; Plin-44::GFP]* | WEN0872 | This paper |
|  | Supplement 2B. | *wenIs0010[Pinx-1::GCaMP6; Punc-122::GFP]; wenEx0873[Pnpr-9::Chrimson::mCherry; Punc-122::RFP]* | WEN0873 | This paper |
| Figure 5—figure supplement 1 | Supplement 1A. | *wenIs0172[Psto-3::GCaMP6::2*NLS::mCardinal; Plin-44::GFP]* | WEN0172 | This paper |
|  | Supplement 1B. | *lite-1(ce314)X; zfIs18[Pmec-4::ChR2::YFP; lin-15+]; wenEx0136[Psto-3::HisCl::UrSL::GFP; Plin-44::GFP]* | WEN0136 | This paper |
|  | Supplement 1C. | *wenEx0567[Pnpr-9::Chrimson::mCherry; Plin-44::GFP; Plim-4(-3328--2174)v1p1::GCaMP6::wCherry]; wenEx0840[Psto-3::miniSOG::UrSL::wCherry; Punc-122::RFP]* | WEN0840 | This paper |
|  | Supplement 1D. | *wenEx0567[Pnpr-9::Chrimson::mCherry; Plin-44::GFP; Plim-4(-3328--2174)v1p1::GCaMP6::wCherry]* | WEN0567 | This paper |
