# Supplementary material for: Flexible motor sequence generation during stereotyped escape responses
Source: eLife. 2020 Jun 5;9:e56942. doi: 10.7554/eLife.56942 (PMC7338056; doi:10.7554/eLife.56942)
Supplement: Supplementary file 2. [file elife-56942-supp2.docx]

**Supplementary File 2. Associated plasmids information**

| **Plasmids** | **Source** | **Identifier** |
| --- | --- | --- |
| *Pacr-5::GCaMP6::2*NLS::mNeptune* | PJH3948 | Zhen Lab |
| *Pacr-5::miniSOG::UrSL::wCherry* | PJH2842 | Zhen Lab |
| *Pavr-14::GFP* | quan0254 | This paper |
| *Pavr-15::GFP* | quan0256 | This paper |
| *Pglc-1::GFP* | quan0209 | This paper |
| *PH-miniSOG* | N/A | Suhong Xu Lab |
| *Pinx-1::mCherry* | quan0118 | This paper |
| *Plgc-55::Chrimson::mCherry* | PJH3743 | Zhen Lab |
| *Plim-4(-3328--2174)v1p1::Arch::GFP* | quan0151 | Alkema Lab |
| *Plim-4(-3328--2174)v1p1::Arch::wCherry* | quan0199 | This paper |
| *Plim-4(-3328--2174)v1p1::GCaMP6::wCherry* | quan0197 | This paper |
| *Plim-4(-3328--2174)v1p1::miniSOG::UrSL::wCherry* | quan0160 | This paper |
| *Plin-44::GFP* | quan0051 | Ge Shan Lab |
| *Pmyo-2::GFP* | quan0052 | Shouhong Guang Lab |
| *Pnmr-1::HisCl::UrSL::GFP* | PJH3746 | Zhen Lab |
| *Pnpr-9::ChR2::GFP* | quan0039 | Alkema Lab |
| *Pnpr-9::Chrimson::mCherry* | quan0074 | This paper |
| *Pnpr-9::eat-4::mCherry* | quan0128 | This paper |
| *Pnpr-9::inx-1a::UrSL::GFP* | quan0220 | This paper |
| *Pnpr-9::inx-1b::UrSL::GFP* | quan0343 | This paper |
| *Pnpr-9::PH-miniSOG::UrSL::wCherry* | quan0234 | This paper |
| *Pnpr-9::TeTx::UrSL::GFP* | quan0453 | This paper |
| *Pnpr-9::TWK-18(gf)::mCherry* | quan0454 | This paper |
| *Prig-3::iGluSnFR* | PJH3683 | Zhen Lab |
| *Psra-11::wCherry* | quan0289 | This paper |
| *Psto-3::Arch::wCherry* | quan0006 | This paper |
| *Psto-3::avr-15a::UrSL::GFP* | quan0352 | This paper |
| *Psto-3::avr-15b::UrSL::GFP* | quan0330 | This paper |
| *Psto-3::avr-15c::UrSL::GFP* | quan0340 | This paper |
| *Psto-3::Chrimson::mCherry* | quan0149 | This paper |
| *Psto-3::GCaMP6::2*NLS::mCardinal* | quan0040 | This paper |
| *Psto-3::HisCl::UrSL::GFP* | quan0089 | This paper |
| *Psto-3::iGluSnFR* | quan0440 | This paper |
| *Psto-3::mCherry* | quan0287 | This paper |
| *Psto-3::miniSOG::UrSL::wCherry* | quan0068 | This paper |
| *Psto-3::TeTx::UrSL::GFP* | quan0601 | This paper |
| *Ptdc-1::Arch::GFP* | quan0084 | This paper |
| *Ptdc-1::ChR2::GFP* | quan0079 | Alkema Lab |
| *Pttx-3::avr-14::UrSL::GFP* | quan0342 | This paper |
| *Pttx-3::glc-1::UrSL::GFP* | quan0354 | This paper |
| *Pttx-3::wCherry* | PJH3236 | Zhen Lab |
| *Punc-122::RFP* | quan0232 | Ge Shan Lab |
| *TeTx* | N/A | Zhengxing Wu Lab |
