# Supplementary material for: Flexible motor sequence generation during stereotyped escape responses
Source: eLife. 2020 Jun 5;9:e56942. doi: 10.7554/eLife.56942 (PMC7338056; doi:10.7554/eLife.56942)
Supplement: Supplementary file 3. [file elife-56942-supp3.docx]

**Supplementary File 3. Associated promoter and gene information**

| **Promoter** | **Primers** | **Length** | **Expression** |
| --- | --- | --- | --- |
|  |  |  |  |
| *lim-4(-3328--2174)p* | AAGCTTTGATTTAGAAATTG | 1.2 kb | RIV, SAA, AWB, SMB |
|  | ACAAGCCGCTCAGTTTTGAT |  |  |
| *inx-1(-898-70)p* | CGCGGATATCAGATTCATAA | 1.0 kb | AIB, AIY (before L2) |
|  | TGTCCACAAAATCGTCGTCGA |  |  |
| *npr-9(-2000-0)p* | GCATGCCAAACAATATAA | 2.0 kb | AIB |
|  | TTCCCAGGAAGTAGCTCTA |  |  |
| *tdc-1(-1459-0)p* | AAGCTTCACCTAACTTCGTC | 1.5 kb | RIM, RIC |
|  | GGATCCTTGGGCGGTCCTGA |  |  |
| *sra-11(-2860--30)p* | CGCACGTTTTCCCTTTAC | 2.8 kb | AIY, AVB, RIF, RIG, AIA |
|  | AATTGTGTGTTGCGGAG |  |  |
| *ttx-3(1076-1909)p* | AAGCTTTTTTGAAACGAT | 0.8 kb | AIY |
|  | ATTTGACACCGAAGACA |  |  |
| *glc-1(-1862-2)p* | TATAGCTAGCAGAAATTGTGATAAGTGG | 1.9 kb | AIY, intestine, pharyngeal and body wall muscle |
|  | TATAGGTACCATTGCCGAAGCAAAAATA |  |  |
| *avr-14(-1487-19)p* | TATAGCATGCCCAAGATACACCCCAGTT | 1.5 kb | AIY, other head neurons and VNC |
|  | TGTAGGTACCGTCAGTCGATAATGCCACA |  |  |
| *avr-15(-3170--19)p* | GTATGCATGCGGCTGTAGAACATCAAAT | 3.1 kb | RIB and other head neurons |
|  | TATAGGTACCTAGAGCAGAACTCCCAAA |  |  |
| *sto-3(-1923-0)p* | CTGCTAGTTCAGCCAAGTCA | 1.9 kb | RIB and unidentified tail neurons |
|  | AAGCCAAACCAAGTGAGAAG |  |  |
| *mec-4(-1020-106)p* | AGCTTCAATACAAGCTC | 1.1 kb | ALM, AVM,PVM, PLM, BDU, FLP, PVD |
|  | CTAGTCGTCTCTTGTAA |  |  |
| **Gene** | **Primers** | **Length** | **Type** |
|  |  |  |  |
| *inx-1a* | TATACCCGGGCGGAATGCTTCTATAT | 1.3 kb | cDNA |
|  | TATAGGTACCTTAGACGAACGTGAAGTAACC |  |  |
| *inx-1b* | TATACCCGGGCGGAATGCTTCTATAT | 1.2 kb | cDNA |
|  | ATAGGTACCTTAGTGGTTGAGGGATTCCGT |  |  |
| *avr-14* | TAGAGGATCCCCGGGATGTGGCATTATCGACTGA | 5.9 kb | genomic DNA |
|  | GGTACCTTAATCACGGCTCTGTTTCACAT |  |  |
| *glc-1* | TATACCCGGGATGGCTACCTGGATTGTC | 2.1 kb | genomic DNA |
|  | TGGAGGTACCCTAAAATAATACGTTCTGCTG |  |  |
| *eat-4* | TATACCCGGGATGTCGTCATGGAACGAGGC | 4.5 kb | genomic DNA |
|  | TATAGGTACCTTCCACTGCTGATAATGCG |  |  |
| *avr-15a* | TTGGCTTATCCCGGGATGATAGGTCGATTGC | 4.0 kb | Minigene |
|  | ATGAGACAGCGGTACCTTACGTACTGATGGCCACAC |  |  |
| *avr-15b* | GGTTTGGCTTATCCCGGGATGTCTACCTCATTTATC | 1.4 kb | cDNA |
|  | ATGAGACAGCGGTACCTTACGTACTGATGGCCACAC |  |  |
| *avr-15c* | TCACTTGGTTTGGCTTATCCCGGGATGTTAGTCATCGTAT | 0.54 kb | cDNA |
|  | ATGAGACAGCGGTACCTTACGTACTGATGGCCACACCGTATT |  |  |
