# Supplementary material for: Flexible motor sequence generation during stereotyped escape responses
Source: eLife. 2020 Jun 5;9:e56942. doi: 10.7554/eLife.56942 (PMC7338056; doi:10.7554/eLife.56942)
Supplement: Supplementary file 4. [file elife-56942-supp4.docx]

**Supplementary File 4. Sample size and replicate number for all experiments**

| **Figure** | **Lable** | **number of worms** | **number of trials (*n*)** |
| --- | --- | --- | --- |
| Figure 1C | P*mec-4::*ChR2 | 73 | 674 |
| Figure 1D | Wild-type | 56 | 385 |
| Figure 1- figure supplement 1A-B and Figure 7C,E | P*mec-4::*ChR2 (no response and pause trials were excluded from data of Figure 1C) | 73 | 594 |
| Figure 2B | P*inx-1*::GCaMP6;P*inx-1*::wCherry | 9 | 59 |
| Figure 2C | P*inx-1*::GCaMP6;P*inx-1*::wCherry | 9 |  |
| Figure 2D | AIB::Chrimson_no ATR | 6 | 43 |
|  | AIB::Chrimson | 8 | 64 |
|  | ALM/AVM::ChR2 | 42 | 284 |
|  | ALM/AVM::ChR2;AIB::Arch | 10 | 49 |
| Figure 2E-F | P*npr-9*::TWK-18(*gf*) | 29 | 332 |
| Figure 2 - figure supplement 1C | P*inx-1*::GCaMP6;P*inx-1*::wCherry | 9 |  |
| Figure 2 - figure supplement 1D | control | 10 | 123 |
|  | 1s | 6 | 101 |
|  | 2s | 8 | 94 |
|  | 3s | 8 | 82 |
|  | 5s | 5 | 61 |
|  | 7s | 7 | 89 |
|  | 12s | 7 | 75 |
|  | 18s | 4 | 50 |
| Figure 2 - figure supplement 1E | control | 12 | 153 |
|  | 1s | 5 | 82 |
|  | 3s | 7 | 105 |
|  | 5s | 7 | 89 |
|  | 7s | 7 | 70 |
|  | 12s | 8 | 74 |
|  | 15s | 7 | 77 |
|  | 18s | 8 | 58 |
| Figure 2 - figure supplement 1F | AIB::Chrimson | 8 | 64 |
|  | AIB::Chrimson;RIM::Arch | 12 | 83 |
|  | RIM::ChR2 | 3 | 30 |
|  | RIM::ChR2;AIB::Arch | 7 | 88 |
| Figure 2 - figure supplement 1G | RIM::ChR2;AIB::TWK-18(*gf*) 1s | 2 | 30 |
|  | RIM::ChR2;AIB::TWK-18(*gf*) 3s | 2 | 42 |
|  | RIM::ChR2;AIB::TWK-18(*gf*) 5s | 2 | 30 |
|  | RIM::ChR2;AIB::TWK-18(*gf*) 7s | 2 | 30 |
|  | RIM::ChR2;AIB::TWK-18(*gf*) 12s | 6 | 32 |
|  | RIM::ChR2 1s | 5 | 82 |
|  | RIM::ChR2 3s | 7 | 105 |
|  | RIM::ChR2 5s | 7 | 89 |
|  | RIM::ChR2 7s | 7 | 70 |
|  | RIM::ChR2 12s | 8 | 74 |
| Figure 2 - figure supplement 1H-I | P*mec-4*::ChR2；AIB::PH-miniSOG | 33 | 308 |
| Figure 3A and Figure 3 - figure supplement 1A | P*lim-4*::GCaMP6 | 5 | 38/18 |
| Figure 3C | control (no ATR) | 6 | 49 |
| Figure 3C and Figure 3 - figure supplement 1C | control (ATR) | 10 | 71 |
| Figure 3C and Figure 3 - figure supplement 1D | *inx-1 unc-9 unc-7* | 6 | 45 |
| Figure 3C and Figure 3 - figure supplement 1E | P*lim-4*::Arch | 5 | 27 |
| Figure 3D | P*npr-9*::Chrimson | 9 | 53 |
|  | *inx-1*;P*npr-9*::Chrimson | 9 | 83 |
|  | *inx-1*;P*npr-9*::Chrimson;P*npr-9*::*inx-1* | 7 | 68 |
| Figure 3E-F and Figure 3 - figure supplement 1F | freely moving | 11 | 44 |
| Figure 3 - figure supplement 1B | P*lim-4*::GCaMP6 (thermal) | 10 | 20 |
| Figure 4A | P*npr-9*::Chrimson | 12 | 85 |
|  | *eat-4* (*ky5*) | 9 | 54 |
|  | *eat-4* (*ky5*)+P*npr-9*::*eat*-4 | 9 | 60 |
|  | P*npr-9*::TeTX | 7 | 44 |
| Figure 4B | P*npr-9*::Chrimson | 8 | 67 |
|  | *avr-14(ad1305) avr-15(vu227) glc-1(pk54)* | 15 | 112 |
|  | triple mutants+P*sto-3*::*avr-15* | 22 | 192 |
|  | triple mutants+P*ttx-3*::*glc-1* | 12 | 119 |
|  | triple mutants+P*ttx-3*::*avr-14* | 12 | 100 |
| Figure 4D and Figure 4 - figure supplement 1C | control (no ATR) | 7 | 52 |
|  | control (ATR) | 8 | 59 |
|  | *eat-4* (*ky5*) | 9 | 69 |
| Figure 4E and Figure 4 - figure supplement 2A | AIB activated; RIB process | 7 | 25 |
|  | *eat-4*;AIB activated; RIB process | 8 | 18 |
|  | AIB activated; RIB cell body | 7 | 12 |
|  | No ATR; RIB Process | 11 | 36 |
| Figure 4 - figure supplement 1B | P*npr-9*::chrimson_no ATR | 6 | 43 |
|  | P*npr-9*:::chrimson | 8 | 64 |
|  | *eat-4*(*ky5*) | 8 | 63 |
|  | *eat-4*(*ky5*)+P*npr-9:::eat-4* | 10 | 67 |
|  | *eat-4(ad572)* | 5 | 52 |
|  | *eat-4(ad572)*+P*npr-9:::eat-4* | 10 | 90 |
|  | *eat-4(ad819)* | 16 | 115 |
|  | *eat-4(ad819)*+P*npr-9:::eat-4* | 7 | 34 |
|  | *avr-14(ad1302)* | 4 | 44 |
|  | *avr-15(ad1051)* | 15 | 105 |
|  | *glc-1(pk54)* | 31 | 233 |
|  | *avr-14(ad1302),avr-15(ad1051)*_strain 1 | 7 | 41 |
|  | *avr-14(ad1302),avr-15(ad1051)*_strain 2 | 8 | 55 |
|  | *avr-15(vu227),glc-1(pk54)* | 3 | 25 |
|  | *avr-14(ad1302),glc-1(pk54)* | 10 | 94 |
| Figure 4 - figure supplement 2B | AIB::Chrimson;AIB::GCaMP6 | 8 | 21 |
| Figure 5A and Figure 5 - figure supplement 1A | type-I transition | 9 | 41 |
|  | type-II transition | 9 | 25 |
| Figure 5B | RIB::Chrimson^1^ | 10 | 65 |
|  | RIB::Chrimson^2^ | 18 | 82 |
|  | RIB::Arch | 14 | 83 |
| Figure 5C | P*mec-4*::ChR2; P*sto-3*::Arch | 30 | 173 |
| Figure 5D-E and Figure 5 - figure supplement 1C | RIV with RIB ablated | 11 | 35 |
|  | RIV calcium activity | 11 | 44 |
| Figure 5F | P*mec-4::*ChR2 | 73 | 594 |
|  | P*mec-4::*ChR2;P*sto-3*::miniSOG | 15 | 77 |
|  | P*mec-4::*ChR2;P*sto-3*::TeTX | 26 | 122 |
| Figure 5 - figure supplement 1B | P*mec-4*::ChR2;P*sto-3*::HisCl | 39 | 313 |
| Figure 5 - figure supplement 1D | SAAD/SMB | 11 | 42 |
|  | RIV | 11 | 44 |
| Figure 6B | AIB::ChR2 | 7 | 49 |
|  | AIB::ChR2;P*lim-4*::Arch | 9 | 73 |
|  | Thermal stimulus | 56 | 385 |
|  | Thermal stimulus;RIV::Arch | 9 | 61 |
|  | Thermal stimulus;P*lim-4*::Arch | 14 | 81 |
| Figure 6C | P*mec-4*::ChR2;P*lim-4*::miniSOG | 34 | 240 |
|  | Wild-type | 30 | 1246 |
